# Digital PCR identifies changes in *CDH1* (E-cadherin) transcription pattern in intestinal-type gastric cancer

**DOI:** 10.18632/oncotarget.13401

**Published:** 2016-11-16

**Authors:** Raefa Abou Khouzam, Chiara Molinari, Samanta Salvi, Monica Marabelli, Valeria Molinaro, Donata Orioli, Luca Saragoni, Paolo Morgagni, Daniele Calistri, Guglielmina Nadia Ranzani

**Affiliations:** ^1^ Department of Biology and Biotechnology, University of Pavia, Pavia, Italy; ^2^ Biosciences Laboratory, Istituto Scientifico Romagnolo per lo Studio e la Cura dei Tumori (IRST) IRCCS, Meldola, Italy; ^3^ Institute of Molecular Genetics, Consiglio Nazionale delle Ricerche, Pavia, Italy; ^4^ Pathology Unit, Morgagni-Pierantoni Hospital, Forlì, Italy; ^5^ Department of General Surgery, Morgagni-Pierantoni Hospital, Forlì, Italy

**Keywords:** CDH1 transcripts, digital-PCR quantification, intestinal-type gastric cancer, normal gastric mucosa

## Abstract

E-cadherin is a cell-cell adhesion protein encoded by *CDH1* tumor-suppressor gene. *CDH1* inactivating mutations, leading to loss of protein expression, are common in gastric cancer of the diffuse histotype, while alternative mechanisms modulating E-cadherin expression characterize the more common intestinal histotype. These mechanisms are still poorly understood. *CDH1* intron 2 has recently emerged as a *cis*-modulator of E-cadherin expression, encoding non-canonical transcripts. One in particular, *CDH1a*, proved to be expressed in gastric cancer cell lines, while being absent in the normal stomach. For the first time, we evaluated by digital PCR the expression of *CDH1* and *CDH1a* transcripts in cancer and normal tissue samples from 32 patients with intestinal-type gastric cancer. We found a significant decrease in *CDH1* expression in tumors compared to normal counterparts (*P* = 0.001), which was especially evident in 76% of cases. *CDH1a* was detected at extremely low levels in 47% of tumors, but not in normal mucosa. A trend was observed of having less *CDH1* in tumors expressing *CDH1a*transcript. The majority of tumors with both a decrease in *CDH1* and presence of *CDH1a* also showed a decrease in *miR-101* expression levels. On the whole, the decrease of *CDH1* transcript, corresponding to the canonical protein, and the presence of *CDH1a*, corresponding to an alternative isoform, are likely to perturb E-cadherin-mediated signaling and cell-cell adhesion, thus contributing to intestinal-type gastric carcinogenesis.

## INTRODUCTION

Gastric cancer (GC) is a heterogeneous disease, with two major histological subtypes, “intestinal” (IGC) and “diffuse” (DGC) [1], that vary in terms of both clinic-pathological profiles and molecular pathogenesis [2]. While IGC follows a stepwise neoplastic progression arising from a premalignant transformation of the normal gastric mucosa, DGC manifests without a defined premalignant stage and is associated with a very aggressive behavior and a poor prognosis [2]. Different genetic and epigenetic lesions underlie the carcinogenic processes involved [2] and subtype-specific molecular signatures have been identified by whole-genome sequencing and gene expression and methylation profiling [3, 4, 5]. Beyond these subtype-specific features, comprehensive molecular approaches also highlighted that adherens junctions and focal adhesions are driver pathways in gastric carcinogenesis, with alterations in genes associated with these pathways occurring in most GC cases [3, 4, 5]. One adhesion-related gene family largely implicated in gastric carcinogenesis is the cadherin family, with the best-known member being *CDH1*.

*CDH1* encodes E-cadherin, a calcium-dependent transmembrane adhesion protein. The downregulation of E-cadherin is a crucial step for epithelial to mesenchymal transition (EMT), whereas the restoration of its expression occurs when the cell phenotype reverts from mesenchymal to epithelial (MET) state. A number of transcription factors and specific activators act along either EMT or MET pathways, tightly regulating E-cadherin expression [6]. Beyond epithelial cell adhesion, the protein has also been implicated in cell survival, proliferation and migration, and its loss/aberrant expression has a key role in tumor invasion and metastasis [7, 8].

Inactivating mutations along the *CDH1* locus leading to loss of protein expression are a common feature of DGCs, while alternative mechanisms modulating E-cadherin expression characterize the intestinal type [9, 10]. A number of studies have shown that *Helicobacter pylori* (*H. pylori*), which is a primary risk factor for IGC, partially mediates the transformation of the normal gastric mucosa by targeting *CDH1* transcription and protein expression levels [8, 11, 12]. Small regulatory RNAs (micro-RNAs) have also been associated with a decrease of E-cadherin expression in IGC; in particular, the loss of miR-101 results in the up-regulation of EZH2, an inhibitor of E-cadherin, thus reducing its expression and promoting tumor progression [13]. In addition to genetic determinants and miRNAs, an intron-mediated mechanism of *CDH1* regulation has also been identified [13, 14, 15]. Indeed, intron 2, harboring an exceptionally high number of repetitive elements involved in exonization, can act as a *cis*-modulator of E-cadherin gene and protein expression [6, 14, 16]. In certain cell lines, intron 2 has been shown to give rise to a number of non-canonical transcripts, one of which, *CDH1a*, harbors properties that enable its translation into a protein isoform differing from the canonical E-cadherin in its N-terminal domain [15]. Such a protein proved to be detectable in transfected cells overexpressing the *CDH1a* transcript; moreover, functional assays associated *CDH1a* overexpression with increased angiogenesis and invasion in the presence of the canonical transcript [15].

These findings make *CDH1* gene transcripts likely players in gastric carcinogenesis of the intestinal type, where some level of E-cadherin expression is often retained. On this basis, we applied digital PCR (dPCR) technique to determine the presence and differential expression of *CDH1* gene transcripts in IGC and normal tissue samples. This represents the first evaluation of the interplay between canonical and non-canonical transcripts of *CDH1* gene in patients affected with GC of the intestinal type.

## RESULTS

We performed *CDH1* gene expression analysis on fresh-frozen tissue samples from 32 patients with gastric cancer of the intestinal type. Available clinical data are reported in Table 1.

**Table 1:** Clinic-pathological parameters of IGC patients

Parameter	Total	
	*n*(32)	%
Sex		
F	14	43.8
M	18	56.2
Age^a^		
≤65	9	28.1
66–75	10	31.3
>75	13	40.6
T^b^		
1	2	6.3
2	17	53.1
3	12	37.5
4	1	3.1
N^b^		
0	12	37.5
1	10	31.3
2	6	18.7
3	4	12.5
M^b^		
0	21	65.6
1	2	6.3
N/A^c^	9	28.1
Grade		
1	1	3.1
2	9	28.1
3	21	65.7
N/A	1	3.1
Tumor site		
Distal third (L)	7	21.9
Middle third (M)	24	75
Proximal third (U)	1	3.1
Tumor size (cm)		
2–5	15	46.9
5–10	15	46.9
>10	1	3.1
N/A	1	3.1
Helicobacter Pylori		
Positive	15	46.9
Negative	15	46.9
N/A	2	6.2
Adjuvant chemotherapy		
Yes	10	31.3
No	16	50
N/A	6	18.7

^a^The mean age at diagnosis was 72.8.

^b^Tumor staging was done based on the tumor (T), lymph node (N) and metastasis (M) system.

^c^N/A: not available.

**Figure 1:**
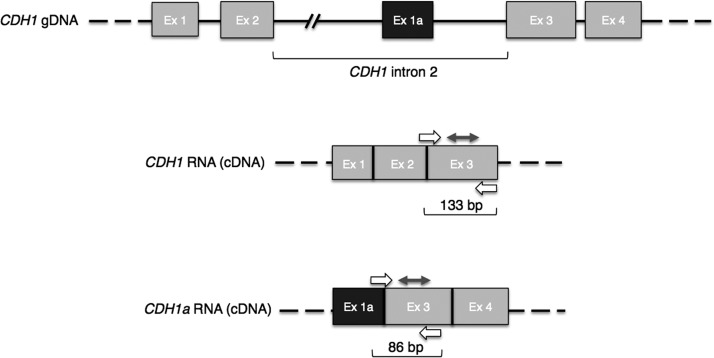
*CDH1* gene and related transcripts In light grey are canonical exons. In dark grey is the *CDH1a* non-canonical exon. Primers and probes designed to detect the specific transcripts are depicted by single and double arrows, respectively; the sizes of resulting amplicons are indicated underneath each transcript. Ex: exon.

Gene expression was investigated by quantifying with digital PCR (dPCR) the canonical transcript (hereafter called *CDH1*) and one non-canonical transcript arising from intron 2 (hereafter called *CDH1a*); this last has properties enabling its translation into a protein isoform differing from the canonical E-cadherin in its N-terminal domain [15]. Figure 1 shows the 5′ end of both *CDH1* and *CDH1a* transcripts, the appropriate primers to obtain these transcripts from total RNA (cDNA), and the specific probes we utilized to quantify them by means of Quantstudio^TM^ 3D dPCR approach.

**Figure 2:**
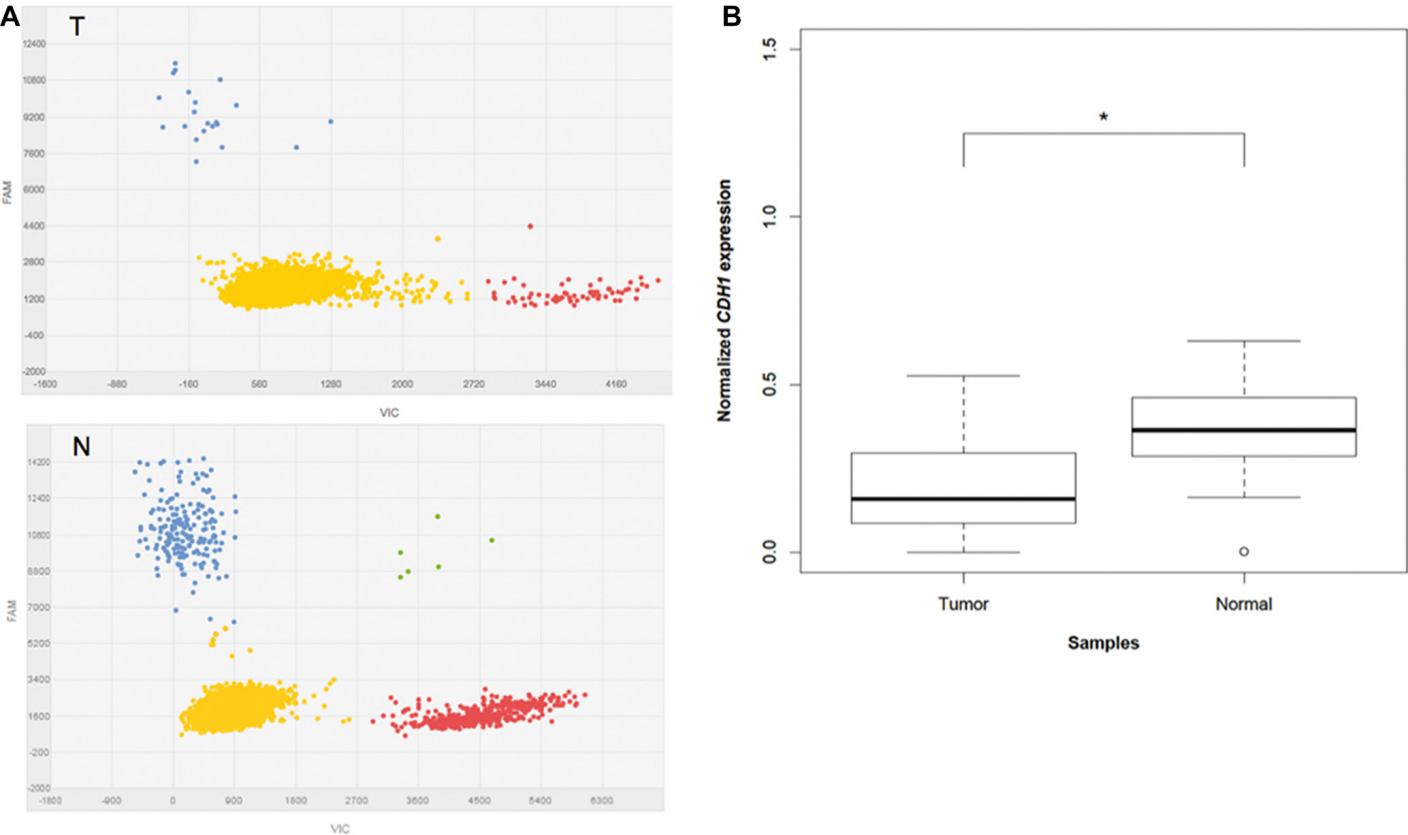
*CDH1* expression analysis in multiplex dPCR (**A**) Typical dPCR output scatter plots of tumor (T) and normal (N) samples showing the distribution of the data points based on the dyes used (VIC and FAM). Yellow refers to “No Amplification”, red to VIC amplified *GAPDH*, blue to FAM amplified *CDH1*, and green to co-amplified *CDH1* and *GAPDH*. (**B**) Box plots of normalized *CDH1* expression levels in 21 tumors compared to the paired normal tissue. “*” refers to a statistically significant difference with a *P-value* = 0.001 as calculated by Student's *t-test*.

By multiplex dPCR, we compared the expression levels of *CDH1* in tumor and corresponding normal tissue samples from 21 IGC patients. Figure 2A shows an example of dPCR output scatter plots obtained for paired samples from the same subject. The analysis of the distribution of *CDH1* expression levels in normal and cancer tissue samples, following normalization to the *GAPDH* reference gene, revealed a significantly lower level of *CDH1* in tumors compared to normal samples (*P* = 0.001) (Figure 2B). In particular, reduced *CDH1* expression by at least 1.5 times was found in 16 out of 21 cases (76%).

**Figure 3:**
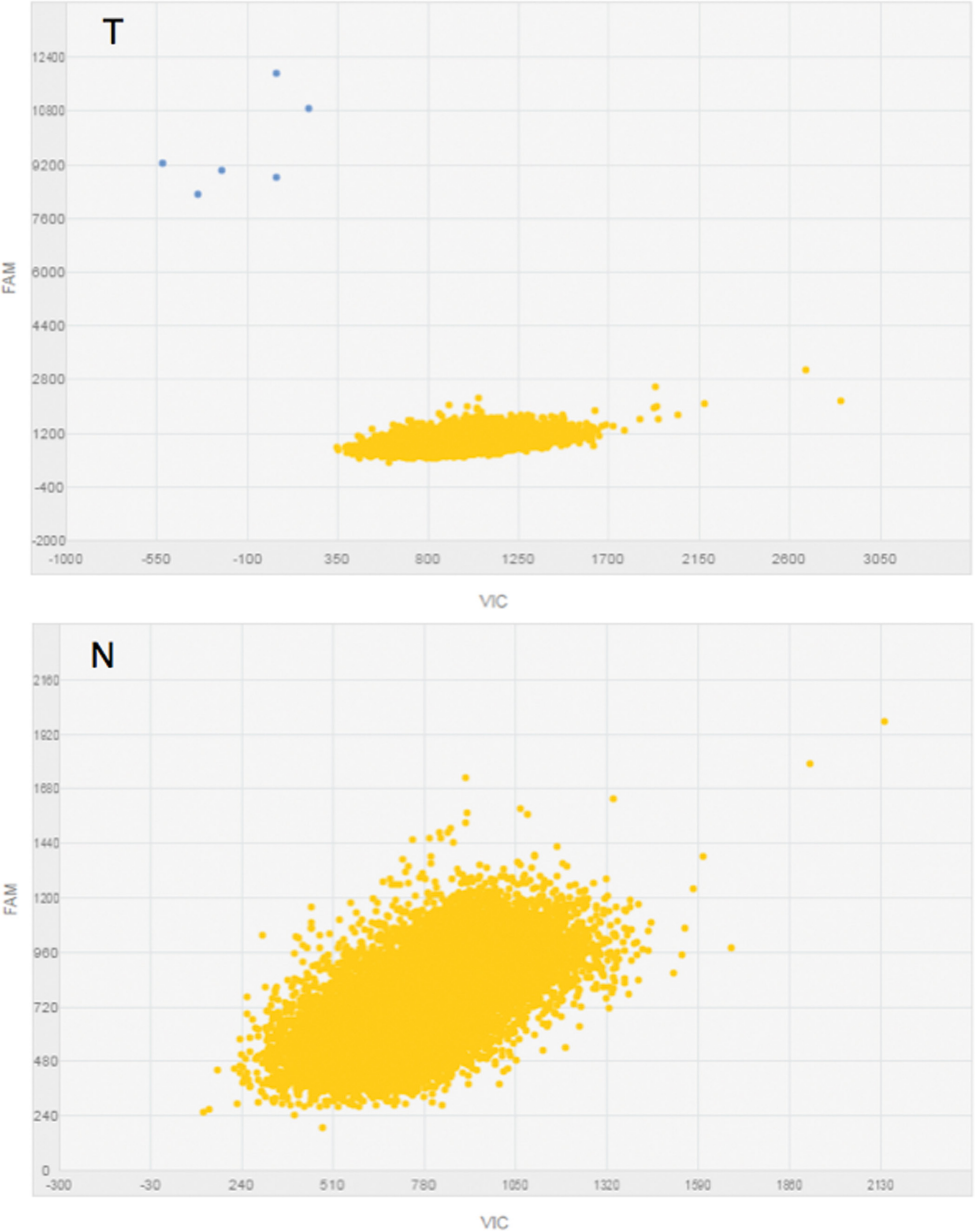
*CDH1a* expression analysis in singleplex dPCR Typical dPCR output scatter plots of tumor (T) and normal (N) samples showing the distribution of data points based on the dye used (FAM). Yellow refers to “No Amplification” and blue to FAM amplified *CDH1a* (see also Appendix S1).

By singleplex dPCR, we then determined the differential expression of *CDH1a* in the 21-paired samples, as well as in 11 additional tumor samples for which the corresponding normal tissue was not available. We could detect *CDH1a* at a very low level in 15 out of 32 (47%) tumors. Under the same experimental conditions, *CDH1a* transcript proved to be undetectable in normal tissue samples, including those corresponding to *CDH1a*-positive tumors. In these tumors, the amount of *CDH1a* was too low to provide for accurate numerical dPCR quantitation; accordingly, we grouped cases as simply being *CDH1a* positive or negative. Figure 3 shows an example of dPCR output scatter plots obtained for paired samples from the same subject, with *CDH1a* being barely detectable in tumor and undetectable in normal cDNA.

**Figure 4:**
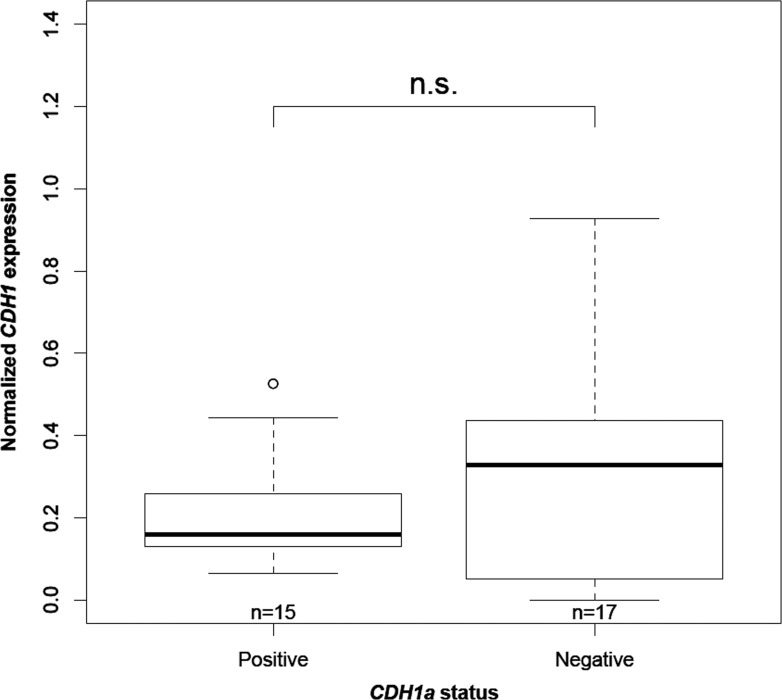
Association between *CDH1* expression and *CDH1a* status “n” is the number of IGC tumors in each category. “n.s” refers to a statistically non-significant difference with a *P-value* = 0.455 as calculated by Wilcoxon rank sum test.

In order to determine whether the expression of the *CDH1a* non-canonical transcript was affecting that of *CDH1*, we compared *CDH1* expression levels in the presence and absence of *CDH1a* in the 32 IGC tumors. A non-statistically significant trend was observed of having more *CDH1* in tumors lacking *CDH1a* (*P* = 0.455) (Figure 4). Moreover, among the 13 tumors showing a decrease in *CDH1* expression levels compared to normal gastric mucosa, 12 (92%) were found to express *CDH1a*, suggesting an association between reduced *CDH1* expression and presence of *CDH1a* in tumors.

**Figure 5:**
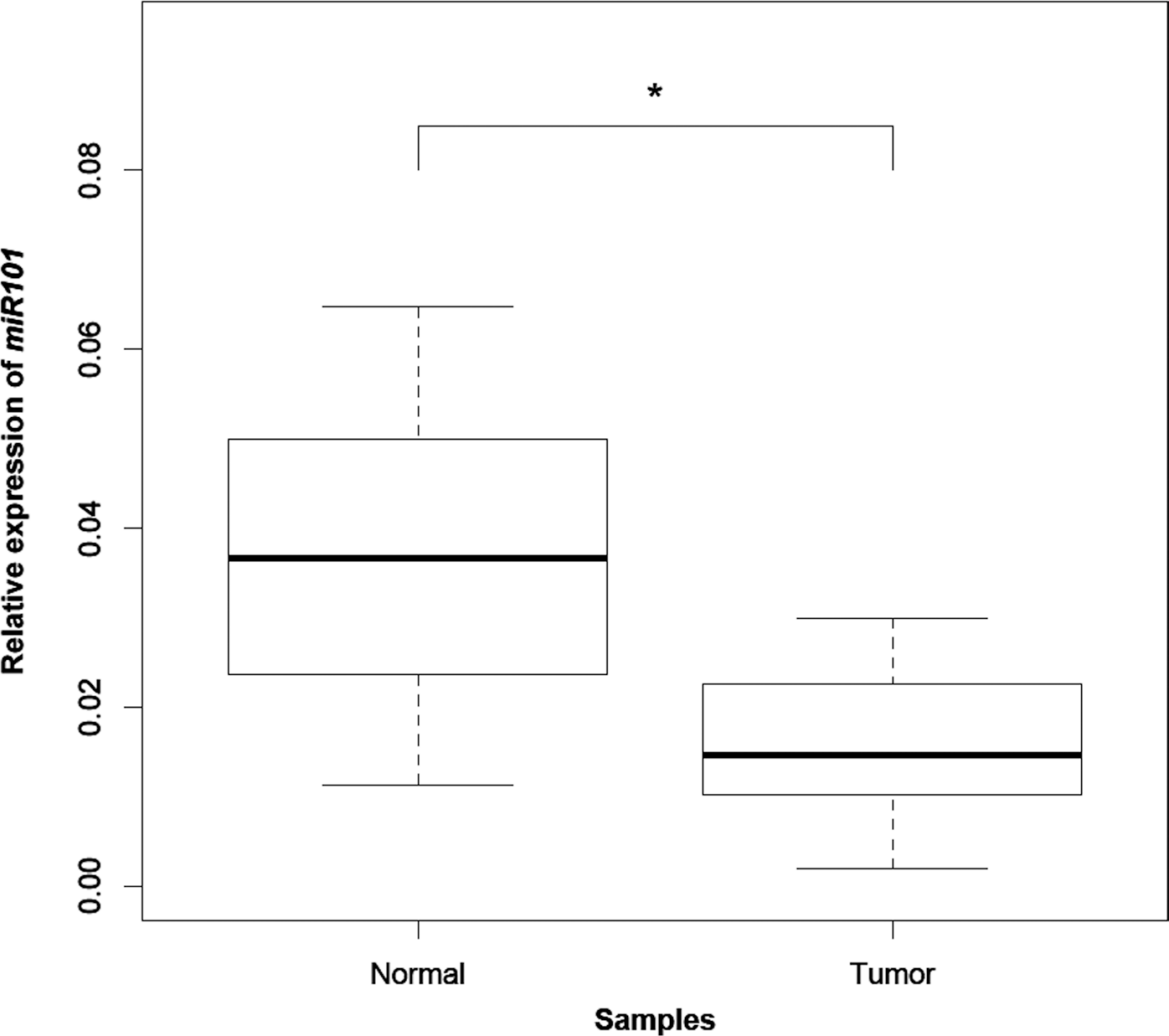
*miR-101* expression analysis by qPCR Box plots of the relative expression of *miR-101* to the internal control gene *RNU6B* in 20 tumors compared to the paired normal tissue. “*” refers to a statistically significant difference with a *P-value* = 1.565 × 10^−05^ as calculated by Wilcoxon rank sum test.

Among the *CDH1* inhibitors, a relevant role is played by EZH2, which is a known target of *miR-101* [13]. The relative quantification of this miRNA in 20 pairs of normal and tumor tissue samples by RT-qPCR showed that *miR-101* significantly decreased in tumors compared to the normal gastric mucosa (*P* = 1.565 × 10^−05^) (Figure 5). Such a decrease proved to be coupled with reduced *CDH1* expression in 13 out of 20 tumors (65%). Moreover, among the 13 tumors harboring a concomitant decrease in both *miR-101* and *CDH1* transcripts, 9 (69%) also expressed *CDH1a*.

It has previously been reported [15] that in GC transfected cells the overexpression of *CDH1a* increases the expression levels of the interferon-inducible *IFITM1* and *IFI27* genes. We applied RT-qPCR method to evaluate *IFITM1* and *IFI27* expression in 15 *CDH1a*-positive and 15 negative tumors: no significant difference in the expression of either *IFITM1* (*P* = 0.486) or *IFI27* (*P* = 0.683) was found in tissue samples.

Finally, we tested for possible associations between *CDH1* expression levels and *CDH1a* presence/absence with patients’ clinical parameters including tumor stage, grade and size, as well as 5-years disease-free survival and presence of *H. pylori*. We found a trend of lower *CDH1* gene expression in tumors with a higher grade (G3 *vs*. G2 + G1) and in those positive for *H. pylori* infection.

## DISCUSSION

The importance of E-cadherin in the manifestation of gastric cancer is highlighted by findings supporting the dysregulation of this protein in both the intestinal and diffuse GC histotypes. Indeed, the majority of GCs show an immunohistochemical (IHC) aberrant pattern (mislocalization) of E-cadherin expression, while complete protein loss is highly predominant in GCs of the diffuse histotype [10, 13, 19]. On the whole, *CDH1* gene alterations account for only about 30–40% of cases with aberrant or absent protein expression, respectively. Among genetic lesions, *CDH1* inactivating mutations are a typical feature of DGC, while promoter hypermethylation and LOH have been found in both tumor histotypes, albeit at different frequencies [10]. Alternative mechanisms have been implicated in the negative regulation of E-cadherin expression, indicating the existence of factors acting at different levels that can subtly modulate E-cadherin expression in gastric cancer of the intestinal type [9, 13].

In this study we evaluated *CDH1* gene expression in the intestinal type by quantifying *CDH1* canonical and *CDH1a* non-canonical transcripts in RNA from normal and cancer tissue samples by means of digital PCR (dPCR). dPCR is a method for sensitive measurement and quantification of nucleic acids; it improves precision and reproducibility with respect to real-time quantitative PCR (qPCR) and provides an alternative approach for detection of gene expression in settings where the target RNA is limited or present in quantity that approaches the limits of qPCR sensitivity [17, 20].

Following dPCR quantification of the canonical transcript, we could demonstrate that *CDH1* was significantly less expressed in cancer tissue compared to normal mucosa of the same patients (*P* = 0.001); this downregulation was especially evident (at least 1.5 times less) in 76% of IGC tumors. Previous evaluations obtained by qPCR method on intestinal GCs gave variable results with reports ranging from a clear decrease [11, 21] to a non-significant difference [22, 23] in *CDH1* expression in cancer compared to the normal counterpart. Therefore, through the implementation of the more precise dPCR in our quantification, we were able to provide evidence on the downregulation of *CDH1* expression.

By applying the same experimental approach, we determined for the first time the expression of *CDH1a* in IGC and could detect *CDH1a* in a fraction of tumors (47%), while no dPCR amplification signal could be observed in the tested normal tissue samples. This finding is in line with data reported by Pinheiro and coworkers [15] who, by Quantitative-SnapShot method, found *CDH1a* to be expressed in gastric cancer cell lines but not in commercially available RNA from the normal stomach. However, while most of their tested cell lines expressed high levels of *CDH1a*, this transcript proved to be barely detectable in the cancer samples we analyzed. A possible explanation is intratumor cell heterogeneity or “dilution” of the *CDH1a* dPCR amplification signal by the presence of normal cells. More likely, *CDH1a* is expressed at a very low level in tumors, while at a high level in cell lines due to positive selection during cell line stabilization.

It has been reported that in transfected GC cell lines, the induced overexpression of *CDH1a* leads to an increase of *IFITM1* and *IFI27* interferon-induced genes [15]. However, in our tissue samples derived from IGC patients, no association was detectable between *CDH1a* presence/absence and *IFITM1* and *IFI27* gene expression levels.

As part of the intricate mechanisms regulating *CDH1*, miRNAs play a role in gene expression dosage, both directly by interacting with the *CDH1* transcript, and indirectly by acting on genes that are part of the regulatory network. In intestinal-type gastric cancer, *miR-101* has been shown to target the *CDH1* inhibitor *EZH2*. In particular, *miR-101* was reported to be downregulated in around 60% of tumors, with a concomitant overexpression of EZH2 and loss/aberrant expression of E-cadherin protein in 40% of cases [13]. By performing *miR-101* RT-qPCR analysis in our paired tumor and normal tissue samples, we could find a significant decrease of expression in tumors compared to the normal counterparts (*P* = 1.565 × 10^−05^). The *miR-101* decrease occurred in 85% of tumors and was accompanied by a concomitant decrease of *CDH1* expression in 65% of cases, thus indicating that *miR-101* might contribute to *CDH1* downregulation. Moreover, 69% of tumors harboring a decrease in both *miR-101* and *CDH1* transcripts expressed *CDH1a*, suggesting a possible link among the three factors.

Besides miRNAs, the presence of *CDH1a* in tumors with lower levels of *CDH1* could be attributed to other mechanisms, including a shift of transcription factors and/or other components of the transcription initiation machinery in favor of one transcript over the other in cancer cells [24]. These types of events, together with disturbance of alternative splicing programs have frequently been linked to the carcinogenic process [25].

In addition, intron 2, from which *CDH1a* arises, is a well-demonstrated *cis* regulatory element of *CDH1* gene expression, containing multiple transcription initiation sites and evolutionary conserved elements, as well as enhancers and repeated sequences [6, 14, 15, 16]. In particular, surrounding exon 1a different regulatory sequences have been identified, including CpG islands, DNaseI hypersensitive sites and CTCF (CCCTC-binding factor) insulator elements [15]. A dysfunction of any of these elements can result in *CDH1* gene transcripts’ imbalance.

Finally, we attempted to associate the expression levels of *CDH1* and the presence/absence of *CDH1a* with the IGC patients’ clinical parameters. We observed a trend of expressing less *CDH1* in higher-grade tumors and in those positive for *H. pylori* infection. While some studies implicated *H. pylori* infection with the epigenetic silencing of *CDH1* promoter [26, 27], others reported alternative mechanisms reducing E-cadherin protein levels through the induction of human E-cadherin-cleaving enzymes [28]. Very recently, it has been shown that *H. pylori* itself secretes a protease (HtrA: high-temperature requirement A) which targets E-cadherin by directly cleaving its extracellular domain, thus opening cell-to-cell junctions [12, 29]. This E-cadherin ectodomain shedding also results in high serum levels of soluble peptides in IGC patients [12, 30].

On the whole, a series of well-known genetic and epigenetic mechanisms can underlie E-cadherin loss or impairment in gastric carcinogenesis. In addition, abnormal isoforms and transcripts’ imbalance resulting from cryptic abnormalities along the *CDH1* locus, may subtly contribute to the carcinogenic process.

## MATERIALS AND METHODS

### Samples

This retrospective study was conducted on 53 fresh-frozen specimens including 21 paired normal/cancer tissue samples and 11 additional tumor tissues. Samples were obtained from 32 patients diagnosed with gastric cancer of the intestinal histotype (Lauren's classification), recruited between 2007 and 2012 at the IRST-IRCCS (Istituto Scientifico Romagnolo per lo Studio e la Cura dei Tumori Srl Istituto di Ricovero e Cura a Carattere Scientifico) of Meldola (FC-Italy). Available clinical data are reported in Table 1. Twenty-two out of 32 patients underwent surgery before 2012; 9 of these patients were disease-free after 5-years of follow up.

Tissue samples were macrodissected from blocks of tumor (containing at least 70% tumor cells) and normal gastric mucosa that had been cryopreserved immediately after surgical resection. The presence of *H. pylori* was further assessed by the examination of hematoxylin and eosin stained formalin-fixed paraffin-embedded tissue sections of each patient.

This study was approved by the Local Ethics Committee (Comitato Etico Area Vasta Romagna e IRST) and informed consent was obtained from all patients (protocol number: IRSTB062).

### RNA extraction

Total RNA was extracted by the TRIzol^®^Reagent (Invitrogen, Darmstadt, Germany) treated with DNase (Qiagen, Hilden, Germany), and purified with the RNeasy MinElute Cleanup Kit (Qiagen) according to the standard protocol provided by the manufacturers. Purified RNA was eluted with RNase free water (Qiagen) and concentration and quality were assessed by Spectrophotometer Nanodrop-ND-1000 (Celbio, Milan, Italy). RNA integrity was verified by using the Experion™ RNA StdSens Analysis Kit (Bio-Rad Laboratories, Hercules, CA, USA) following manufacturer's instructions (Supplementary Figure S1).

### Reverse transcription (RT)

RT of 1 μg RNA was carried out using the iScript™ cDNA Synthesis Kit (Bio-Rad Laboratories) according to manufacturer's instructions. Thermal cycling conditions were as follows: 25°C for 5 min, 42°C for 30 min, 85°C for 5 min. The resulting cDNA was either immediately analyzed by dPCR or qPCR or stored at −20°C.

### Digital PCR (dPCR)

dPCR was used to determine the expression of *CDH1* and *CDH1a* with *GAPDH* as an internal control (Appendix S1). All dPCR experiments were carried out using the chip-based QuantStudio^TM^ 3D Digital PCR system (Applied Biosystems, Foster City, CA, USA), in accordance with the “Minimum Information for Publication of Quantitative Real-Time dPCR Experiments” (dMIQE) guidelines (Supplementary Table S1) [17]. *CDH1* and *GAPDH* reactions were run in multiplex using 10–20 ng cDNA, while *CDH1a* reactions were run in singleplex using 300 ng cDNA (Appendix S1, Supplementary Figure S2). In each case, reaction mixes containing either cDNA or water (no-template controls) were first prepared by adding 2X QuantStudio 3D™ Digital PCR Master Mix v2 (Applied Biosystems) and 20X gene specific assay (Supplementary Table S2) in a total volume of 15.5 μl. Fifteen μl of each sample were then loaded into a blade that is firmly clasped onto the arm of the chip loader and evenly distributed into the chip's 20 000 nano-sized reaction wells. Each chip was then coated with Immersion Fluid and sealed with a QuantStudio™ 3D Digital PCR Chip Lid v2. Up to 24 chips were run simultaneously using GeneAmp PCR System 9700 (Applied Biosystems) by applying the following conditions: hold at 96°C for 10 min; 45 cycles of 60°C for 2 min and 98°C for 30 sec; hold at 60°C for 2 min. At the end of the reaction, chips were processed using the QuantStudio™ 3D Digital PCR system (Applied Biosystems) and analyzed with QuantStudio™ 3D Analysis Suite™ software (version 3.0.3). Only chips with at least 13 000 analyzable data points were accepted (Supplementary Figure S3). The data were exported as a comma-separated values (CSV) file and included the number of copies per μl of each target in the tested sample.

### Two-step reverse transcription quantitative PCR (RT-qPCR)

To quantify *IFI27* and *IFITM1* levels, RT-qPCR experiments were performed in triplicate on 20 ng of tumor cDNA obtained from the previously described RT. qPCR reactions were carried out using PrimeTime^®^ Std qPCR assays for the target genes *IFI27* and *IFITM1* (Integrated DNA Technologies, Leuven, Belgium) and the endogenous controls, *RPLP0* and *HPRT1* (Integrated DNA Technologies, Coralville, IA, USA), together with the TaqMan^®^ Universal Master Mix II, no UNG (Applied Biosystems).

With respect to the quantification of *miR-101* transcript, 10 ng total RNA from normal and tumor tissue samples were first reverse transcribed using RT-specific primers and components of the TaqMan^®^ MicroRNA Reverse Transcription Kit (Applied Biosystems), according to the manufacturer's instructions. Subsequently, qPCR reactions were performed in triplicate for the mature *miR-101* transcript and for an endogenous control, *RNU6B*, with TaqMan^®^ Universal Master Mix II, no UNG (Applied Biosystems), using the TaqMan™MicroRNA Assays (Applied Biosystems) has-miR-101 and RNU6B, respectively.

All aforementioned qPCR reactions were run on a 7500 Real-Time PCR (Applied Biosystems) by applying the following thermal cycling protocol: hold at 95°C for 10 min; 40 cycles of 95°C for 30 sec and 60°C for 60 sec; and quantified using the comparative 2^−ΔCt^ method [18].

### Statistical analysis

To compare mRNA expression either a two-tailed Student's *t-test* or a two-sided Wilcoxon rank-sum test was used depending on the data distribution. With respect to associations between clinic-pathological parameters and mRNA expression, two-sided Wilcoxon rank-sum test and Fisher's exact tests were used. All analysis was done with R statistical software version 2.14.1. *P*-values ≤ 0.05 were considered as statistically significant for each comparison.

## SUPPLEMENTARY MATERIALS FIGURES AND TABLES




