# Supplementary material for: Digital PCR identifies changes in CDH1 (E-cadherin) transcription pattern in intestinal-type gastric cancer
Source: Oncotarget. 2016 Nov 16;8(12):18811–20. doi: 10.18632/oncotarget.13401 (PMC5386649; doi:10.18632/oncotarget.13401)
Supplement: Supplementary file 1 [file oncotarget-08-18811-s001.pdf]

Digital PCR identifies changes in *CDH1* (E-cadherin) transcription pattern in intestinal-type gastric cancer

Supplementary Materials

Appendix S1 dPCR set up and calculations

Chip based digital PCR is an end-point reaction that relies on the calibrated partitioning of a sample into thousands of wells, such that each well contains zero or a single target molecule. Amplification then occurs only in the wells containing a copy of the target and is indicated by a florescent signal. The absolute number of target molecules in the original sample can then be calculated by determining the ratio of positive to total partitions using binomial Poisson statistics. In this way dPCR does not require the use of standards or internal controls. However, in the case of gene expression analysis, variability can be introduced in a number of steps required to attain cDNA from the original RNA. Therefore, in our analysis we included *GAPDH* as an endogenous expression control to normalize for such variability as it is expressed at levels comparable to the target *CDH1*.

To ensure the most accurate copy number calculation, various amounts of cDNA were tested for *CDH1* and *CDH1a* expression, either alone (singleplex dPCR) or in combination with *GAPDH* (multiplex dPCR). As a result, *CDH1* and *GAPDH* could be successfully multiplexed using 10 or 20 ng of cDNA, depending on the sample. In the case of *CDH1a* that is expressed at very low levels, we used the maximum amount of cDNA to ensure amplification, which in our case was 300 ng. This amount was up to 30 times more than that required for accurate copy number quantification of *GAPDH*, indicating that *CDH1a* dPCR reactions must be done in singleplex.

At the end of the *CDH1a* singleplex and *CDH1* and *GAPDH* multiplex dPCR reactions, the absolute amounts of the transcripts in each sample were calculated by multiplying the reported “number of copies per µl” with the loaded reaction volume (15 µl) and dividing the resulting value by the initial amount of cDNA. The absolute values of *GAPDH* were subsequently used to normalize both *CDH1a* and *CDH1* expression in the tested tissue, since all three transcripts were derived from the same reverse transcription of the same RNA, extracted from each sample.

The feasibility of the multiplex dPCR reaction was assessed by comparing normalized *CDH1* expression levels in a couple of samples in singleplex and multiplex settings. The resulting amounts of *CDH1* were quite similar in the two settings (coefficient of variation (CV) = 5%), which allowed us to proceed with multiplexing.

Moreover, we tested the repeatability of our method by performing intra-assay dPCR replicates of 4 randomly selected samples. The average CV was found to be 10%.

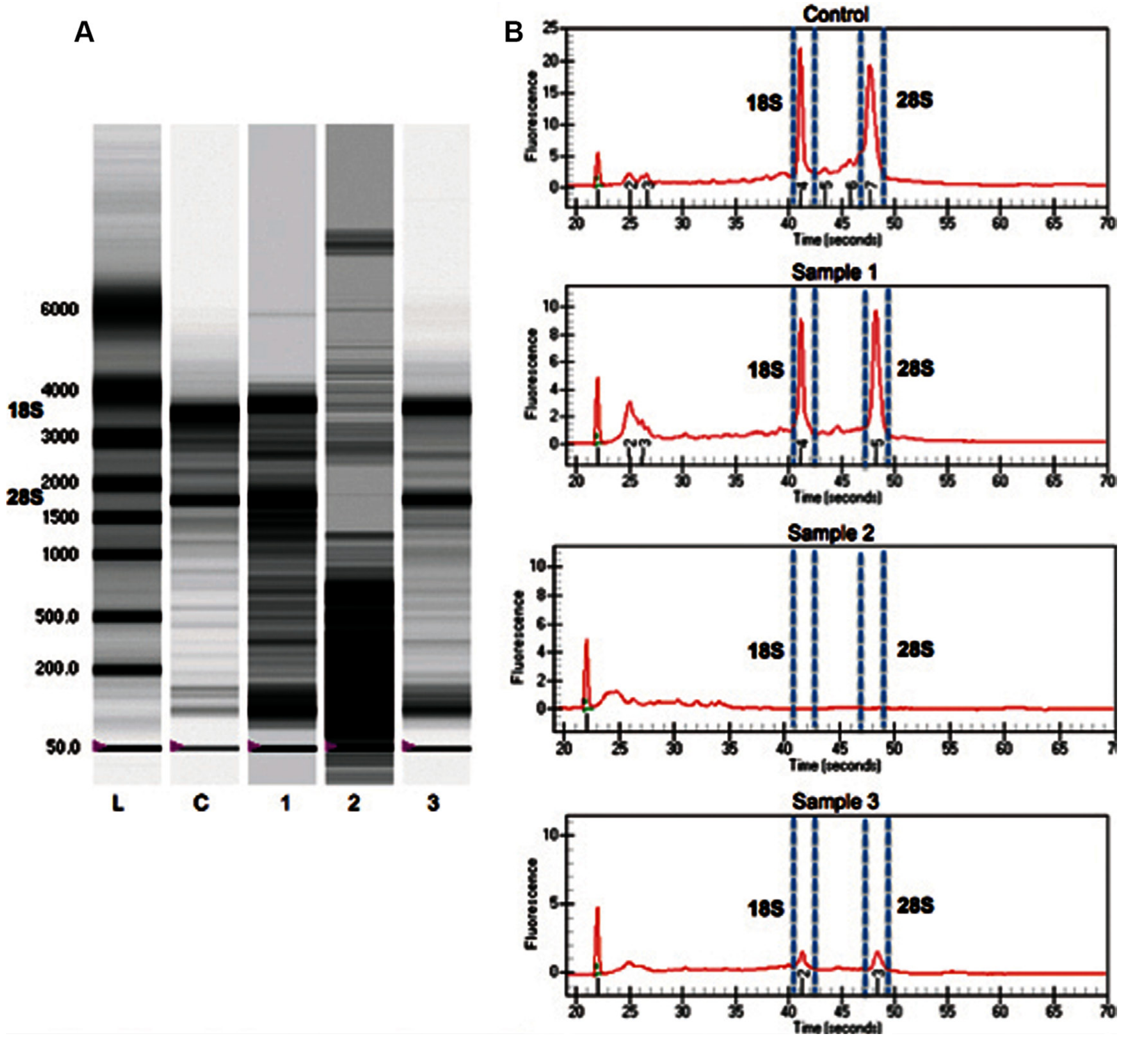

Supplementary Figure S1: Experion results. (**A**) Gel electrophoresis of Experion RNA ladder (L), Experion total RNA control (C) and RNA derived from 3 IGC patients (1, 2, 3). (**B**) Electropherogram of the Experion total RNA control and the three patients. The relative positions of the 18S rRNA and 28S rRNA are indicated. Only samples showing clear peaks for the two rRNAs that are comparable with the control RNA were selected. In this example, sample 2 was excluded.

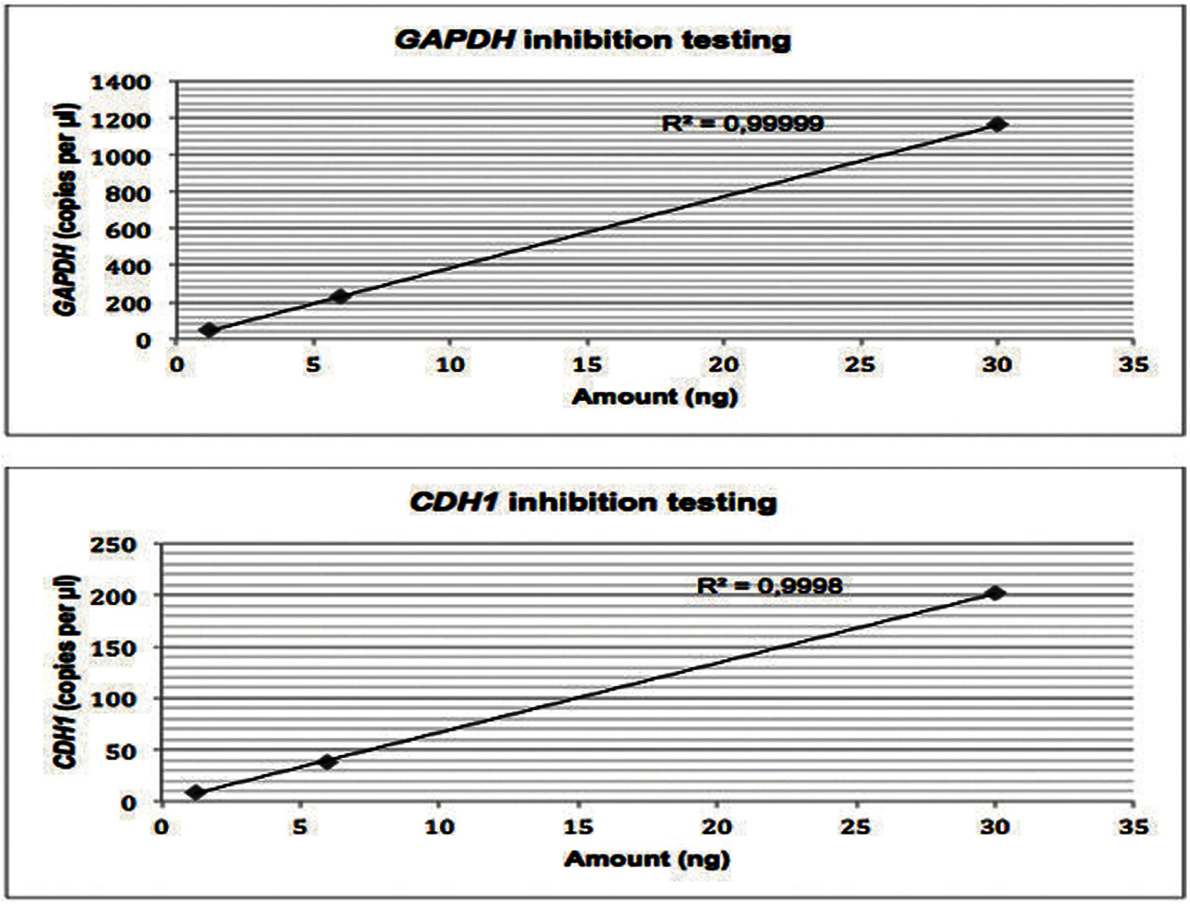

Supplementary Figure S2: dPCR inhibition testing. A 1:5 serial dilutions were performed on the cDNA of a randomly selected sample to test for the effect of inhibitors on dPCR. The number of copies per µl was obtained from the dPCR reactions for each dilution. There is an almost perfect correlation between the added amount of cDNA (ng) and the reported number of copies per µl (correlation coefficient R^2 is almost 1), indicating that inhibitors do not affect the reaction.

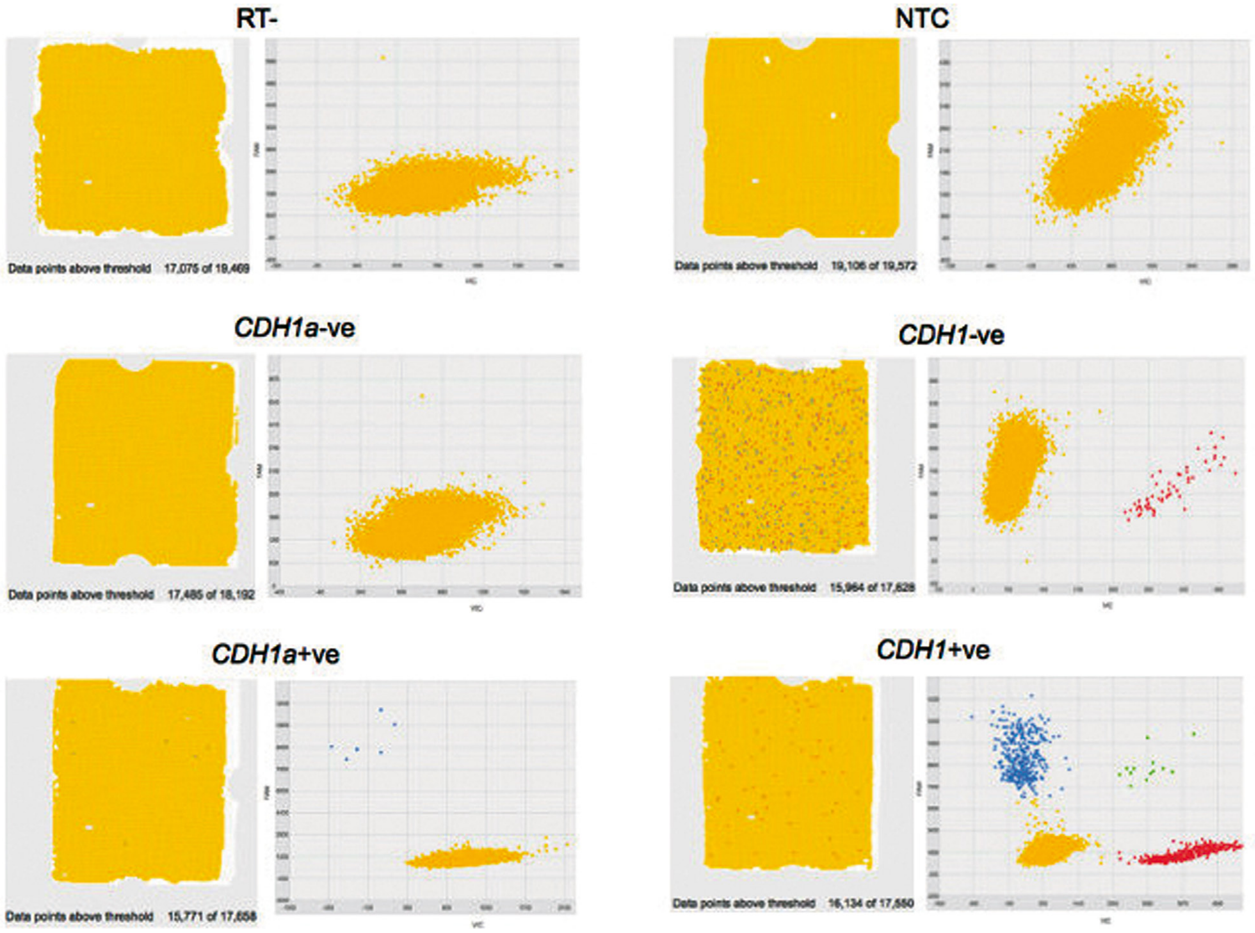

Supplementary Figure S3: Typical dPCR results. For each sample, on the left side is the chip view, which shows the wells and the number of analyzable data points. In our analysis we only accepted samples with at least 13 000 data points above threshold. On the right side is the scatter plot showing the distribution of the data points based on the dyes used (VIC and FAM). Yellow refers to "No Amplification", red to the VIC amplified GAPDH, blue to the FAM amplified *CDH1* or *CDH1a*, and green to co-amplified *CDH1* and *GAPDH*. RT- refers to a sample reverse transcribed without including the reverse transcriptase, serving as a negative control for the RT reaction. NTC refers to a no-template control in which water is added instead of cDNA, serving as a negative control for the dPCR reaction. *CDH1a*-ve and *CDH1*-ve are examples of a sample that is negative for both transcripts. *CDH1a*+ve and *CDH1*+ve are examples of a sample that is positive for both transcripts.

Supplementary Table S1: dMIQE checklist for authors, reviewers and editors.
See Supplementary_Table_S1

Supplementary Table S2: dPCR assays

Assay	Target	Accession number[b]	Primer/Probe Sequences and modifications (5′ to 3′)[d]	Location	Amplicon size (bp)
CDH1 IDT[a] custom designed assay	*CDH1*	NM_004360	(F) GTCCTGGGCAGAGTGAATTT<br>(R) GTGGGTTATGAAACCGTAGAGG<br>(P)/FAM/TCAAAGTGGGCACAGATG GTGTGA/TAMRA/	Exon 2-3<br>Exon 3<br>Exon 3	133
CDH1a IDT custom designed assay	*CDH1a*	N/A[c]	(F) GCTGCAGTTTCACTTTTAGTG<br>(R) ACTTTGAATCGGGTGTCGAG<br>(P)/FAM/CGGTCGACAAAGGACA GCCTATT/TAMRA/	Intron 2-Exon 3<br>Exon 3<br>Exon 3	86
Hs.PT.39a.22214836 PrimeTime® Std qPCR Assay	*GAPDH*	NM_002046	Manufacturer's Propriety	Manufacturer's Propriety	Manufacturer's Propriety

[a]IDT: Integrated DNA Technologies.
[b]Accession number is based on the NCBI gene website.
[c]N/A: Not available; *CDH1a* is a novel non-canonical transcript without an accession number and its provided location is based on the accession number of the canonical transcript (*CDH1*).
[d]F: forward primer; R: reverse primer; P probe; FAM: Fluorescein; TAMRA: Carboxytetramethylrhodamine; dPCR optimized assay concentrations: 900 nM (F), 900 nM (R), 250 nM (P).
