# Supplementary material for: Digital PCR identifies changes in CDH1 (E-cadherin) transcription pattern in intestinal-type gastric cancer
Source: Oncotarget. 2016 Nov 16;8(12):18811–20. doi: 10.18632/oncotarget.13401 (PMC5386649; doi:10.18632/oncotarget.13401)
Supplement: Supplementary file 2 [file oncotarget-08-18811-s002.doc]

**Table S1** dMIQE checklist for authors, reviewers and editors.

| **ITEM TO CHECK** | **IMPORTANCE^a^** | **COMMENTS/**  **WHERE? ^b^** |
| --- | --- | --- |
| **EXPERIMENTAL DESIGN** | | |
| Definition of experimental and control groups | **E** | Materials and Methods |
| Number within each group | **E** | Materials and Methods |
| Assay carried out by core lab or investigator's lab? | D | Investigator’s lab |
| Acknowledgement of authors' contributions | D | Author Contributions |
| **SAMPLE** | | |
| Description | **E** | Materials and Methods |
| Volume/mass of sample processed | D | Available on request |
| Microdissection or macrodissection | **E** | Materials and Methods |
| Processing procedure | **E** | Materials and Methods |
| If frozen - how and how quickly? | **E** | Materials and Methods |
| If fixed - with what, how quickly? | **E** | Not Applicable |
| Sample storage conditions and duration (especially for FFPE samples) | **E** | Materials and Methods |
| **NUCLEIC ACID EXTRACTION** | | |
| Procedure and/or instrumentation | **E** | Materials and Methods |
| Name of kit and details of any modifications | **E** | Materials and Methods |
| Source of additional reagents used | D | Materials and Methods |
| Details of DNase or RNase treatment | **E** | Materials and Methods |
| Contamination assessment (DNA) | **E** | DNase treatment |
| Nucleic acid quantification | **E** | Materials and Methods |
| Instrument and method | **E** | Materials and Methods |
| Purity (A260/A280) | D | Available on request |
| Yield | D | Available on request |
| RNA integrity method/instrument | **E** | Materials and Methods;  Figure S1 |
| Inhibition testing (Cq dilutions, spike or other) | **E** | Figure S2 |
| **REVERSE TRANSCRIPTION** | | |
| Complete reaction conditions | **E** | Materials and Methods |
| Amount of RNA and reaction volume | **E** | Materials and Methods |
| Priming oligonucleotide (if using GSP) and concentration | **E** | Not Applicable |
| Reverse transcriptase and concentration | **E** | Materials and Methods;  Manufacturer’s proprietary |
| Temperature and time | **E** | Materials and Methods |
| Manufacturer of reagents and catalogue numbers | D | Manufacturer: Materials and Methods |
| Estimated copies measured with and without RT | D | Figure S3 |
| Storage conditions of cDNA | D | Materials and Methods |
| **dPCR TARGET INFORMATION** | | |
| Sequence accession number | **E** | Table S2 |
| Location of amplicon | D | Table S2 |
| Amplicon length | **E** | Table S2 |
| *In silico* specificity screen (BLAST, etc.) | **E** | Available on request |
| Pseudogenes, retropseudogenes or other homologs? | D | None detected by BLAST |
| Sequence alignment | D | Available on request |
| Secondary structure analysis of amplicon | D | Available on request |
| Location of each primer by exon or intron | **E** | Table S2 |
| What splice variants are targeted? | **E** | Table S2 |
| **dPCR OLIGONUCLEOTIDES** | | |
| Primer sequences | **E** | Table S2 |
| RTPrimerDB Identification Number | D | Not Applicable |
| Probe sequences | D | Table S2 |
| Location and identity of any modifications | **E** | Table S2 |
| Manufacturer of oligonucleotides | D | Materials and Methods |
| Purification method | D | HPLC |
| **dPCR PROTOCOL** | | |
| Complete reaction conditions | **E** | Materials and Methods |
| Reaction volume and amount of cDNA/DNA | **E** | Materials and Methods |
| Primer, (probe), Mg++ and dNTP concentrations | **E** | Materials and Methods; Manufacturers’ proprietary |
| Polymerase identity and concentration | **E** | Materials and Methods |
| Buffer/kit identity and manufacturer | **E** | Materials and Methods |
| Exact chemical constitution of the buffer | D | Manufacturers’ proprietary |
| Additives (SYBR Green I, DMSO, etc.) | **E** | None |
| Manufacturer of plates/tubes and catalogue number | D | Available on request |
| Complete thermocycling parameters | **E** | Materials and Methods |
| Reaction setup (manual/robotic) | D | Manual setup |
| Gravimetric or volumetric dilutions (manual/robotic) | D | Manual volumetric |
| Total PCR reaction volume prepared | D | Materials and Methods |
| Partition number | **E** | 20 000 |
| Individual partition volume | **E** | 755 pL |
| Total volume of the partitions measured (effective reaction size) | **E** | Number of partitions multiplied by 755 pL |
| Partition volume variance/SD | D | Not Applicable |
| Comprehensive details and appropriate use of controls | **E** | Appendix S1;  Figure S3 |
| Manufacturer of dPCR instrument | **E** | Materials and Methods |
| **dPCR VALIDATION** | | |
| Optimization data for the assay | D | Available on request |
| Specificity (gel, sequence, melt, or digest) | **E** | Not possible after digital PCR |
| Limit of detection of calibration control | D | N/A |
| If multiplexing, comparison with singleplex assays | **E** | Appendix S1 |
| **DATA ANALYSIS** | | |
| Mean copies per partition (λ or equivalent) | **E** | Not Applicable |
| dPCR analysis program (source, version) | **E** | Materials and Methods |
| Outlier identification and disposition | **E** | No outliers identified |
| Results of NTCs | **E** | Figure S3 |
| Examples of positive(s) and negative experimental results as supplemental data | **E** | Figure S3 |
| Justification of number and choice of reference genes | **E** | Appendix S1 |
| Description of normalization method | **E** | Appendix S1 |
| Number and concordance of biological replicates | D | N/A |
| Number and stage (RT or dPCR) of technical replicates | **E** | Appendix S1 |
| Repeatability (intra-assay variation) | **E** | Appendix S1 |
| Reproducibility (inter-assay variation, %CV) | D | N/A |
| Experimental variance or CI | D | N/A |
| Statistical methods for result significance | **E** | Materials and Methods |
| Software (source, version) | **E** | Materials and Methods |
| Cq or raw data submission using RDML | D | Not Applicable |

^a^ E: essential information; D: desirable information.

^b^ N/A: not available.
